# Hospital Festivals, Meetings, &c.

**Published:** 1894-03-03

**Authors:** 


### HOSPITAL FESTIVALS, MEETINGS, ETC.

### THE MIDDLESEX HOSPITAL.

A quarterly general court of governors was held at the Middlesex Hospital on February 22nd, Mr. Henry N. Custance presiding. In moving the adoption of the report, Lord Sandhurst announced that, on April 13th, H.R.H. the Prince of Wales would take the chair at the annual dinner in aid of the funds of the hospital at the Hôtel Métropole. The chairman then briefly referred to the deaths of Mr. R. Ruthven Pym, who had been treasurer to the hospital for twenty-one years, and of Mr. W. F. Lucas, one of the medical officers, from diphtheria, contracted while assisting at an operation. He was glad to be able to report that land had now been secured for a convalescent home at Clacton-on-Sea. Mr. John B. Sedgwick seconded the adoption of the report, and the resolution was carried.

### NATIONAL DENTAL HOSPITAL.

The new building of the National Dental Hospital was opened on Saturday, February 24th, by H.R.H. the Duke of York, who was accompanied by the Duke of Fife and Prince Edward of Saxe-Weimar. This new hospital has been erected at a cost of about £10,000 by the Dowager Lady Howard de Walden, after designs by Mr. A. E. Thompson, and contains a lecture hall, laboratories, demonstration room, and accommodation for 75 patients. In declaring the hospital open the Duke of York spoke of the useful work being done by the institution, and hoped nothing might interfere with the ends in view—the alleviation of dental troubles amongst the suffering poor and the education of a race of future dental surgeons who may do honour to their special branch of surgery.

### HOSPITAL SATURDAY FUND.

On the afternoon of Saturday, February 24th, the Lord Mayor presided over the annual meeting of the Hospital Saturday Fund in the Egyptian Hall of the Mansion House, there being a crowded audience. Amongst others on the platform were Cardinal Vaughan, Lord Sandhurst, Sir J. Colomb, Mr. Reginald Acland (chairman of the Council), Mr. Hamilton Hoare, and Mr. W. Bousfield. The report of the committee stated that the actual receipts for the year were £19,544, against £20,309 for 1892, in addition to which there was brought forward a balance of £1,113. The expenses of the year, after eliminating all charges of the nature of awards, were £2,564, against £2,444 for the year 1892. The amount distributed for 1893 was £17,778, and a balance of £315 had been carried forward. Mr. R. B. Acland, the President, said the year 1893 had been the best ever known in the history of the fund. There had been distributed to the various institutions over £750 more than in any previous year. From the street and workshop collections they received in coppers no less than £1,500 in one day. The number of letters issued for hospitals and convalescent homes amounted to 24,896, which represented an immense amount of good done. There had during the year been sent to convalescent homes on the coast 359 persons, who had contributed 55 per cent. of the cost of the railway journey and their stay. That was a development of the fund started during the past two years. Cardinal Vaughan moved, "That this meeting approves of the principle of soliciting small weekly subscriptions in aid of the metropolitan medical charities from those engaged in workshops, warehouses, and places of business in the metropolis." He congratulated the meeting on the magnificent work which was being carried out by the working people of London. The street collection formed noble testimony to the generous hearts of the English working men. Mr. W. Bousfield seconded the motion, which was carried unanimously. Lord Sandhurst moved, "That the meeting cordially approves of the efforts of the Hospital Saturday Fund to increase the interest of the working classes in the work of the metropolitan medical institutions." In expressing an opinion that the working classes should be represented on the boards of management of the hospitals, he pointed out, however, that no one could successfully fill such a position unless he had a considerable amount of leisure, and a person who had to work hard for weekly wages could not give the necessary time. Admiral Sir J. Colomb, M.P., seconded the motion, and a vote of thanks to the Lord Mayor closed the proceedings.

### WEST LONDON HOSPITAL.

The report presented to the general meeting of governors on February 26th recorded a large decrease in the receipts of this hospital, the income having fallen from £11,932 in 1892 to £6,721 in 1893, the difference being attributed largely to a decrease in legacies. Subscriptions having come in but slowly, the building fund for the further extension of the hospital has not yet reached the required amount. The sum necessary for the erection of the first additional block, to contain about sixty more beds, is about £15,000, and such an increase to the resources of the hospital is most urgently needed in the neighbourhood. The meeting unanimously accepted the resolution proposed by the Rev. Canon Graham, D.D., that a petition should be presented praying Her Majesty the Queen to grant to the hospital a royal charter of incorporation, enabling the institution to hold real as well as personal property. The Committee of Management were unanimously re-elected, with the exception of Dr. Irvine Menzies, who resigned his post, and the proceedings came to an end with a vote of thanks to the chairman, W. Bird, Esq., J.P., D.L.

### THE ROYAL NATIONAL HOSPITAL FOR CONSUMPTION AND DISEASES OF THE CHEST, VENTNOR.

The annual general meeting of Governors of this hospital was held on Thursday, February 22nd, 1894, at the London office, 34, Craven Street, Charing Cross, Sir Richard E. Webster, Q.C., M.P., Chairman of the Board of Management, presiding in the absence of the President, the Earl of Rosebery, K.G. The report for the past year referred to the loss sustained by the lamented death of the late chairman, Mr. Herbert C. Saunders, Q.C., and stated that the Board had been happy in securing the kind co-operation of Sir Richard Webster as a colleague, and had elected Sir Richard Webster their chairman in the place of the late Mr. H. C. Saunders, Q.C. The number of patients treated had been larger than in any previous year; 790 in-patients having been under medical treatment in 1893, of whom 31 died; the average length of stay of each patient having been 54 days. The total cash receipts during the past year amounted to £10,091 5s. 5d., and the total expenditure to £11,358 11s. 6d. In addition, the sons of the late Mr. Crumpton John Nunn, who was for twenty years a member of the Board, generously presented the hospital with stock value £1,000, for the perpetual endowment of one bed in their father's memory. It is hoped that other friends will adopt this appropriate way of commemorating the memory of a deceased relative or friend. The need of a spire to complete the chapel having been pointed out by the founder, Dr. Hassall, the Board gladly gave him permission to issue an appeal for the necessary funds. In the medical section of the report, Dr. Sinclair Coghill, F.R.C.P., Senior Physician to the hospital, advanced the theory of the infectious, as opposed to the hereditary, nature of consumption. The report was adopted. The re-election of officers and a vote of thanks to the chairman brought the proceedings to a close.

### GREAT NORTHERN CENTRAL HOSPITAL.

The annual meeting of the General Council of Governors of the above hospital took place on Friday, February 23rd, with Mr. C. T. Murdoch in the chair. During the past year the building of the hospital has been completed, and as soon as funds permit of it the number of beds can be raised from 70 to 160. For the present, the financial condition of the institution will only allow of the use of 120. The chairman laid stress on the necessity for clearing off the debt of £15,000 which has been necessitated by the building operations, alluding to the falling off of the income of the hospital during the past year, owing partly to no legacies having been received. He also expressed the pleasure which had been given to the committee by the Duke of York's acceptance of the office of President of the hospital. Allusion was made by several speakers to the untiring services rendered by Dr. Cholmeley, retiring Hon. Physician, and one of the original founders of the hospital, whom, as a mark of their appreciation of his work, the Governors have appointed Consulting Physician. The loss which the hospital had sustained in the death of Sir Andrew Clark was also alluded to. Mr. B. L. Cohen, M.P., proposed a vote of thanks to the Ladies' Association headed by Mrs. Harkness, for their assistance in the work of the hospital. The Ladies' Association has already collected one sum of £5,000 towards the building and maintenance of the institution, and, for the purpose of raising a second such sum, proposes to hold a bazaar on April 18th, 19th, and 20th, which will be presided over by H.R.H. the Duchess of Teck.

### ROYAL FREE HOSPITAL

At the annual court of governors of the Royal Free Hospital, Gray's Inn Road, held on February 22nd, the chair was taken by W. Tarn Pritchard, Esq., whose address chiefly related to the building of a new front to the hospital, at present proceeding, and for which £5,000 of the needful £12,000 has been already subscribed. He was followed by Mr. North, who announced that, although three wards of the hospital had been closed during the alterations, yet, by increased economy of space, the number of in-patients received had been only 54 less than in 1892, while the number of out-patients continued to increase. He laid great stress on the economical management of the hospital, and said it would be well if the public were aware that the debt for the building of the new front had not been incurred until the state of the hospital rendered the alterations an absolute necessity. Mr. Burt then spoke of the unreliable nature of the income of the hospital, the regular subscriptions providing but a comparatively small part, and hoped that by the time the new buildings were opened the debt upon them would have been cleared off. Reference was then made to the valuable services rendered to the hospital in the past by the hon. treasurer, Edward Masterman, Esq., who has been forced by failing health to resign his post. His place is taken by W. Tarn Pritchard, Esq.

### SOCIETY FOR THE PROTECTION OF BIRDS.

The annual meeting of the above society took place at 105, Jermyn Street, on February 22nd, and was very well attended. Sir Herbert Maxwell, Bart., M.P., who was expected to preside, was detained by business, and the chair was therefore taken by E. H. Bayley, Esq., M.P. Letters regretting absence were also received from the Duchess of Portland, the Earl of Onslow, Viscount Wolseley, Lord Lilford, and others. The Chairman, in his introductory speech, alluded to the evils resulting from the extermination of various species of wild birds from an industrial as well as a humanitarian point of view, and urged the need of such an extension of the Wild Birds' Protection Act as should ensure every species of wild bird being left unmolested during certain months of the year. Mr. Lemon then read extracts from the report, which chronicled a remarkable increase in the number of members of the society, there being now 9,159 as against 5,200 last year. The adoption of the report was then moved by Mrs. Phillips, who referred to the short time their society had been in existence, and characterised the movement as, at any rate at the beginning, a woman's movement. She hoped that, as time went on, their "young society" would gain experience in overcoming the many difficulties that lay in the way of a widespread acceptance of its principles.

